# Methyl 2-(2-{[(benz­yloxy)carbon­yl]amino}­propan-2-yl)-5-hy­droxy-6-oxo-1,6-dihydro­pyrimidine-4-carboxyl­ate

**DOI:** 10.1107/S1600536811016278

**Published:** 2011-05-07

**Authors:** Zhenhua Shang, Jing Ha, Yifeng Yu, Xiaodan Zhao

**Affiliations:** a College of Chemical and Pharmaceutical Engineering, Hebei University of Science and Technology, Shijiazhuang 050018, People’s Republic of China

## Abstract

In the title compound, C_17_H_19_N_3_O_6_, the dihedral angle between the two aromatic rings is 45.9 (1)°. The crystal structure is stabilized through inter­molecular N—H⋯O hydrogen bonds and intra­molecular O—H⋯O hydrogen bonds are also present.

## Related literature

For related structures, see: Fun *et al.* (2009 ▶); Shang & Shang (2007 ▶). The title compound is an inter­mediate in the preparation of the anti­retroviral drug raltegravir [systematic name *N*-(2-(4-(4-fluoro­benzyl­carbamo­yl)-5-hy­droxy-1-meth­yl-6-oxo-1,6-dihydro­pyrimidin-2-yl)propan-2-yl)-5-methyl-1,3,4-oxadiazole-2-carboxamide. For therapeutic details of raltegravir, see Steigbigel *et al.* (2008 ▶). For synthetic details, see: Culbertson (1979 ▶).
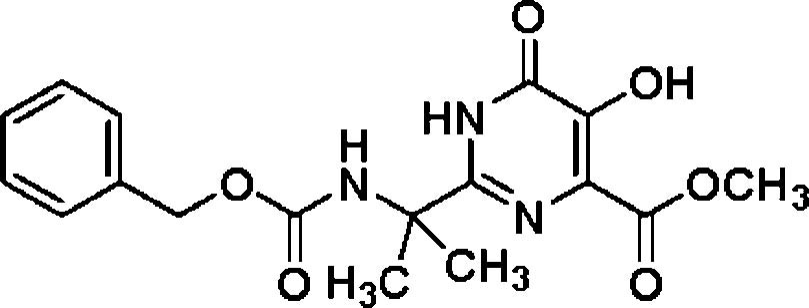


## Experimental

### 

#### Crystal data

C_17_H_19_N_3_O_6_
*M* *_r_* = 361.35
Monoclinic, 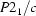

*a* = 12.122 (2) Å
*b* = 16.300 (3) Å
*c* = 9.1766 (18) Å
β = 106.29 (3)°
*V* = 1740.4 (6) Å^3^
*Z* = 4
Mo *K*α radiation
μ = 0.11 mm^−1^
*T* = 113 K
0.24 × 0.20 × 0.16 mm

#### Data collection

Bruker SMART diffractometer
Absorption correction: multi-scan (*SADABS*; Bruker, 1997 ▶) *T*_min_ = 0.975, *T*_max_ = 0.983
15564 measured reflections
4142 independent reflections
3386 reflections with *I* > 2σ(*I*)
*R*_int_ = 0.035

#### Refinement

*R*[*F* ^2^ > 2σ(*F* ^2^)] = 0.039
*wR*(*F* ^2^) = 0.103
*S* = 1.09
4142 reflections
250 parameters
H atoms treated by a mixture of independent and constrained refinement
Δρ_max_ = 0.33 e Å^−3^
Δρ_min_ = −0.24 e Å^−3^

### 

Data collection: *SMART* (Bruker, 1997 ▶); cell refinement: *SAINT* (Bruker, 1997 ▶); data reduction: *SAINT*; program(s) used to solve structure: *SHELXS97* (Sheldrick, 2008 ▶); program(s) used to refine structure: *SHELXL97* (Sheldrick, 2008 ▶); molecular graphics: *SHELXTL* (Sheldrick, 2008 ▶); software used to prepare material for publication: *SHELXTL*.

## Supplementary Material

Crystal structure: contains datablocks global, I. DOI: 10.1107/S1600536811016278/wn2426sup1.cif

Structure factors: contains datablocks I. DOI: 10.1107/S1600536811016278/wn2426Isup2.hkl

Supplementary material file. DOI: 10.1107/S1600536811016278/wn2426Isup3.cml

Additional supplementary materials:  crystallographic information; 3D view; checkCIF report

## Figures and Tables

**Table 1:** Hydrogen-bond geometry (Å, °)

*D*—H⋯*A*	*D*—H	H⋯*A*	*D*⋯*A*	*D*—H⋯*A*
N3—H3⋯O5^i^	0.882 (15)	2.133 (15)	2.8911 (14)	143.7 (12)
N2—H2⋯O2^ii^	0.938 (16)	1.886 (16)	2.8135 (16)	169.3 (13)
O1—H1⋯O3	0.918 (17)	1.788 (17)	2.6163 (14)	148.7 (16)

Symmetry codes: (i) 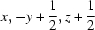; (ii) 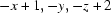.

**Fig. 1.**
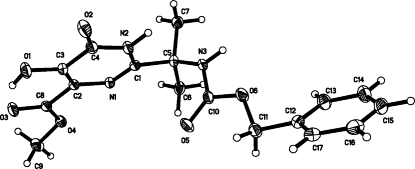
The molecular structure of the title compound, drawn with 30% probability ellipsoids. Hydrogen atoms are shown as spheres of arbitrary radius.

**Fig. 2.**
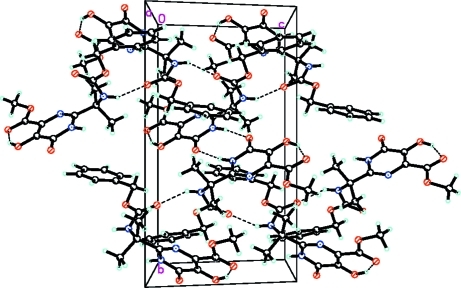
The packing of the title compound,viewed down the a axis. The dashed lines indicate the hydrogen bonds.

**Fig. 3.**
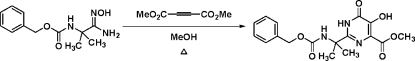
The formation of the title compound.

## supplementary crystallographic information

### Comment

Raltegravir (MK-0518, brand name Isentress), an antiretroviral drug produced by Merck & Co, is used to treat HIV infection (Steigbigel *et al.*, 2008). It received FDA approval in October 2007, the first of a new class of HIV drugs, the integrase inhibitors, to receive such approval. The title compound is a key intermediate in the preparation of Raltegravir.

The pyrimidinone ring is planar, as it is in a related compound (Fun *et al.*, 2009). This is in contrast with another related compound (Shang *et al.*, 2007), where the heterocyclic ring is twisted. In the title compound the dihedral angle between the two aromatic rings is 45.9 (1)°. The crystal structure is stabilized through intermolecular N—H···O hydrogen bonds; intramolecular O—H···O hydrogen bonds are also present.

### Experimental

The title compound was prepared by a published method (Culbertson, 1979). To a slurry of benzyl 1-amino-1-(hydroxyimino)-2-methylpropan-2-ylcarbamate (2.9 g) in methanol (12 ml) was added dimethyl acetylenedicarboxylate (1.77 g) slowly at room temperature. After 1.5 h, the mixture was added to xylene (20 ml). The reaction mixture was then heated to reflux for 2 h and cooled to 60 °C. Methyl tert-butyl ether (9 ml) was added slowly to build a seed bed. The batch was then cooled to 0 °C for 14 h, and then further cooled to -5 °C and allowed to stand for 1 h before filtration. The solid was washed with methyl tert-butyl ether (4 ml) and dried. 50 mg of the title compound was dissolved in 30 ml methanol and the solution was kept at room temperature for 10 d. Natural evaporation gave colorless single crystals of the title compound which were  suitable for X-ray analysis.

### Refinement

All H atoms attached to C atoms were positioned geometrically and treated as riding with C—H = 0.95 Å (aromatic), 0.98 Å (methyl group) and 0.99 Å (methyene group). *U*_iso_(H) = x*U*_eq_(C), where x = 1.5 for methyl H and 1.2 for all other carbon-bound H atoms. The positional parameters of the nitrogen-bound H and oxygen-bound H atoms were refined freely (N—H = 0.882 (15) and 0.938 (16) Å; O—H = 0.918 (17) Å).

### Crystal data

**Table:**

C_17_H_19_N_3_O_6_	*D*_x_ = 1.379 Mg m^−^^3^
*M**_r_* = 361.35	Melting point = 183–185 K
Monoclinic, *P*2_1_/*c*	Mo *K*α radiation, λ = 0.71073 Å
*a* = 12.122 (2) Å	Cell parameters from 4990 reflections
*b* = 16.300 (3) Å	θ = 2.5–27.9°
*c* = 9.1766 (18) Å	µ = 0.11 mm^−^^1^
β = 106.29 (3)°	*T* = 113 K
*V* = 1740.4 (6) Å^3^	Plate, colorless
*Z* = 4	0.24 × 0.20 × 0.16 mm
*F*(000) = 760	

### Data collection

**Table:**

Bruker SMART diffractometer	4142 independent reflections
Radiation source: rotating anode	3386 reflections with *I* > 2σ(*I*)
confocal	*R*_int_ = 0.035
ω scans	θ_max_ = 27.9°, θ_min_ = 2.5°
Absorption correction: multi-scan (*SADABS*; Bruker, 1997)	*h* = −15→15
*T*_min_ = 0.975, *T*_max_ = 0.983	*k* = −21→18
15564 measured reflections	*l* = −11→12

### Refinement

**Table:**

Refinement on *F*^2^	Primary atom site location: structure-invariant direct methods
Least-squares matrix: full	Secondary atom site location: difference Fourier map
*R*[*F*^2^ > 2σ(*F*^2^)] = 0.039	Hydrogen site location: inferred from neighbouring sites
*wR*(*F*^2^) = 0.103	H atoms treated by a mixture of independent and constrained refinement
*S* = 1.09	*w* = 1/[σ^2^(*F*_o_^2^) + (0.0539*P*)^2^ + 0.1964*P*] where *P* = (*F*_o_^2^ + 2*F*_c_^2^)/3
4142 reflections	(Δ/σ)_max_ = 0.001
250 parameters	Δρ_max_ = 0.33 e Å^−^^3^
0 restraints	Δρ_min_ = −0.23 e Å^−^^3^

### Special details

**Table:**

Experimental. 1*H*-NMR (500 MHz, DMSO) 1.51(s, 6H), 3.82(s, 3H), 4.98(s, 2H), 7.35(bs, 5H), 7.45 (s, 1H), 10.24(s, 1H), 12.58(s, 1H)
Geometry. All e.s.d.'s (except the e.s.d. in the dihedral angle between two l.s. planes) are estimated using the full covariance matrix. The cell e.s.d.'s are taken into account individually in the estimation of e.s.d.'s in distances, angles and torsion angles; correlations between e.s.d.'s in cell parameters are only used when they are defined by crystal symmetry. An approximate (isotropic) treatment of cell e.s.d.'s is used for estimating e.s.d.'s involving l.s. planes.
Refinement. Refinement of *F*^2^ against ALL reflections. The weighted *R*-factor *wR* and goodness of fit *S* are based on *F*^2^, conventional *R*-factors *R* are based on *F*, with *F* set to zero for negative *F*^2^. The threshold expression of *F*^2^ > σ(*F*^2^) is used only for calculating *R*-factors(gt) *etc*. and is not relevant to the choice of reflections for refinement. *R*-factors based on *F*^2^ are statistically about twice as large as those based on *F*, and *R*- factors based on ALL data will be even larger.

### Fractional atomic coordinates and isotropic or equivalent isotropic displacement parameters (Å^2^)

**Table:**

	*x*	*y*	*z*	*U*_iso_*/*U*_eq_	
N1	0.18567 (8)	0.11941 (5)	0.81459 (10)	0.0159 (2)	
N2	0.36822 (8)	0.06461 (6)	0.92786 (11)	0.0179 (2)	
N3	0.39582 (8)	0.18366 (6)	1.15086 (11)	0.0170 (2)	
O1	0.28432 (8)	−0.00917 (5)	0.54614 (9)	0.0247 (2)	
O2	0.45987 (7)	−0.02265 (5)	0.80609 (9)	0.0282 (2)	
O3	0.08230 (7)	0.05655 (5)	0.42611 (9)	0.0234 (2)	
O4	0.01176 (7)	0.14406 (5)	0.56692 (9)	0.01951 (19)	
O5	0.40819 (7)	0.26902 (6)	0.95868 (9)	0.0279 (2)	
O6	0.54363 (7)	0.26725 (5)	1.18631 (10)	0.0285 (2)	
C1	0.27523 (9)	0.11186 (6)	0.93025 (12)	0.0148 (2)	
C2	0.18776 (9)	0.07855 (6)	0.68378 (12)	0.0160 (2)	
C3	0.27744 (10)	0.03116 (7)	0.67123 (12)	0.0176 (2)	
C4	0.37656 (10)	0.02098 (7)	0.80389 (13)	0.0196 (2)	
C5	0.27947 (9)	0.15246 (6)	1.08134 (12)	0.0157 (2)	
C6	0.18968 (10)	0.22037 (7)	1.05981 (14)	0.0215 (3)	
H6A	0.1929	0.2448	1.1585	0.032*	
H6B	0.1130	0.1973	1.0147	0.032*	
H6C	0.2055	0.2626	0.9924	0.032*	
C7	0.25766 (11)	0.08712 (7)	1.18957 (13)	0.0216 (3)	
H7A	0.3156	0.0437	1.2026	0.032*	
H7B	0.1810	0.0635	1.1472	0.032*	
H7C	0.2624	0.1122	1.2882	0.032*	
C8	0.08872 (9)	0.09093 (7)	0.54685 (12)	0.0171 (2)	
C9	−0.07754 (10)	0.16603 (8)	0.43090 (14)	0.0259 (3)	
H9A	−0.0423	0.1845	0.3526	0.039*	
H9B	−0.1245	0.2103	0.4542	0.039*	
H9C	−0.1262	0.1181	0.3938	0.039*	
C10	0.44448 (10)	0.24266 (7)	1.08715 (13)	0.0185 (2)	
C11	0.60365 (11)	0.33419 (8)	1.13972 (14)	0.0266 (3)	
H11A	0.5518	0.3818	1.1074	0.032*	
H11B	0.6333	0.3173	1.0541	0.032*	
C12	0.70133 (10)	0.35578 (7)	1.27658 (13)	0.0206 (3)	
C13	0.68387 (11)	0.36043 (8)	1.41951 (14)	0.0247 (3)	
H13	0.6100	0.3491	1.4314	0.030*	
C14	0.77345 (11)	0.38150 (8)	1.54519 (15)	0.0284 (3)	
H14	0.7609	0.3838	1.6427	0.034*	
C15	0.88144 (11)	0.39923 (8)	1.52900 (16)	0.0302 (3)	
H15	0.9424	0.4144	1.6149	0.036*	
C16	0.89961 (11)	0.39465 (8)	1.38730 (16)	0.0282 (3)	
H16	0.9733	0.4067	1.3755	0.034*	
C17	0.80992 (10)	0.37236 (7)	1.26174 (15)	0.0230 (3)	
H17	0.8232	0.3685	1.1648	0.028*	
H3	0.4221 (12)	0.1800 (8)	1.2505 (17)	0.024 (3)*	
H2	0.4269 (13)	0.0577 (9)	1.0186 (18)	0.036 (4)*	
H1	0.2178 (15)	0.0049 (10)	0.474 (2)	0.045 (5)*	

### Atomic displacement parameters (Å^2^)

**Table:**

	*U*^11^	*U*^22^	*U*^33^	*U*^12^	*U*^13^	*U*^23^
N1	0.0170 (5)	0.0159 (4)	0.0139 (4)	−0.0015 (3)	0.0029 (4)	−0.0008 (3)
N2	0.0177 (5)	0.0210 (5)	0.0134 (4)	0.0034 (4)	0.0014 (4)	−0.0024 (4)
N3	0.0182 (5)	0.0189 (5)	0.0116 (4)	−0.0021 (4)	0.0004 (4)	−0.0005 (4)
O1	0.0292 (5)	0.0293 (5)	0.0143 (4)	0.0056 (4)	0.0038 (4)	−0.0051 (3)
O2	0.0265 (5)	0.0372 (5)	0.0188 (4)	0.0140 (4)	0.0029 (4)	−0.0049 (4)
O3	0.0259 (5)	0.0283 (4)	0.0135 (4)	−0.0018 (3)	0.0012 (3)	−0.0026 (3)
O4	0.0173 (4)	0.0220 (4)	0.0158 (4)	0.0007 (3)	−0.0010 (3)	0.0012 (3)
O5	0.0294 (5)	0.0371 (5)	0.0142 (4)	−0.0095 (4)	0.0009 (3)	0.0056 (4)
O6	0.0273 (5)	0.0334 (5)	0.0189 (4)	−0.0152 (4)	−0.0031 (4)	0.0055 (4)
C1	0.0154 (5)	0.0143 (5)	0.0147 (5)	−0.0011 (4)	0.0040 (4)	0.0004 (4)
C2	0.0180 (6)	0.0156 (5)	0.0129 (5)	−0.0018 (4)	0.0019 (4)	0.0005 (4)
C3	0.0227 (6)	0.0164 (5)	0.0130 (5)	−0.0011 (4)	0.0037 (4)	−0.0015 (4)
C4	0.0220 (6)	0.0202 (6)	0.0158 (5)	0.0029 (4)	0.0040 (4)	−0.0011 (4)
C5	0.0158 (5)	0.0169 (5)	0.0135 (5)	−0.0011 (4)	0.0025 (4)	−0.0028 (4)
C6	0.0209 (6)	0.0212 (6)	0.0209 (6)	0.0033 (4)	0.0034 (5)	−0.0051 (5)
C7	0.0265 (6)	0.0216 (6)	0.0182 (6)	−0.0037 (4)	0.0087 (5)	−0.0015 (4)
C8	0.0183 (6)	0.0171 (5)	0.0149 (5)	−0.0043 (4)	0.0031 (4)	0.0009 (4)
C9	0.0209 (6)	0.0308 (7)	0.0201 (6)	0.0011 (5)	−0.0037 (5)	0.0048 (5)
C10	0.0197 (6)	0.0209 (5)	0.0139 (5)	−0.0017 (4)	0.0030 (4)	−0.0018 (4)
C11	0.0276 (7)	0.0319 (7)	0.0181 (6)	−0.0124 (5)	0.0028 (5)	0.0028 (5)
C12	0.0208 (6)	0.0190 (5)	0.0204 (6)	−0.0025 (4)	0.0034 (5)	0.0002 (4)
C13	0.0195 (6)	0.0305 (6)	0.0230 (6)	−0.0005 (5)	0.0041 (5)	−0.0011 (5)
C14	0.0306 (7)	0.0309 (7)	0.0206 (6)	0.0027 (5)	0.0020 (5)	−0.0040 (5)
C15	0.0239 (6)	0.0263 (6)	0.0317 (7)	−0.0014 (5)	−0.0067 (5)	0.0005 (5)
C16	0.0172 (6)	0.0249 (6)	0.0390 (7)	−0.0007 (5)	0.0019 (5)	0.0080 (6)
C17	0.0236 (6)	0.0202 (6)	0.0262 (6)	0.0016 (4)	0.0084 (5)	0.0042 (5)

### Geometric parameters (Å, °)

**Table:**

N1—C1	1.2937 (14)	C6—H6A	0.9800
N1—C2	1.3792 (14)	C6—H6B	0.9800
N2—C4	1.3692 (15)	C6—H6C	0.9800
N2—C1	1.3704 (14)	C7—H7A	0.9800
N2—H2	0.938 (16)	C7—H7B	0.9800
N3—C10	1.3446 (15)	C7—H7C	0.9800
N3—C5	1.4663 (14)	C9—H9A	0.9800
N3—H3	0.882 (15)	C9—H9B	0.9800
O1—C3	1.3456 (14)	C9—H9C	0.9800
O1—H1	0.918 (17)	C11—C12	1.5062 (16)
O2—C4	1.2308 (14)	C11—H11A	0.9900
O3—C8	1.2245 (14)	C11—H11B	0.9900
O4—C8	1.3230 (14)	C12—C17	1.3874 (18)
O4—C9	1.4491 (13)	C12—C13	1.3881 (18)
O5—C10	1.2154 (14)	C13—C14	1.3872 (17)
O6—C10	1.3489 (13)	C13—H13	0.9500
O6—C11	1.4413 (14)	C14—C15	1.389 (2)
C1—C5	1.5242 (15)	C14—H14	0.9500
C2—C3	1.3646 (16)	C15—C16	1.380 (2)
C2—C8	1.4881 (15)	C15—H15	0.9500
C3—C4	1.4602 (16)	C16—C17	1.3924 (18)
C5—C6	1.5259 (15)	C16—H16	0.9500
C5—C7	1.5291 (16)	C17—H17	0.9500
			
C1—N1—C2	116.87 (10)	H7A—C7—H7C	109.5
C4—N2—C1	123.91 (10)	H7B—C7—H7C	109.5
C4—N2—H2	117.4 (9)	O3—C8—O4	123.86 (10)
C1—N2—H2	118.4 (9)	O3—C8—C2	122.24 (11)
C10—N3—C5	123.00 (9)	O4—C8—C2	113.87 (9)
C10—N3—H3	115.1 (9)	O4—C9—H9A	109.5
C5—N3—H3	116.8 (9)	O4—C9—H9B	109.5
C3—O1—H1	104.1 (11)	H9A—C9—H9B	109.5
C8—O4—C9	115.30 (9)	O4—C9—H9C	109.5
C10—O6—C11	116.95 (9)	H9A—C9—H9C	109.5
N1—C1—N2	123.03 (10)	H9B—C9—H9C	109.5
N1—C1—C5	120.75 (10)	O5—C10—N3	126.23 (11)
N2—C1—C5	116.14 (9)	O5—C10—O6	124.12 (11)
C3—C2—N1	123.81 (10)	N3—C10—O6	109.62 (9)
C3—C2—C8	118.60 (10)	O6—C11—C12	105.89 (9)
N1—C2—C8	117.51 (10)	O6—C11—H11A	110.6
O1—C3—C2	126.16 (10)	C12—C11—H11A	110.6
O1—C3—C4	114.98 (10)	O6—C11—H11B	110.6
C2—C3—C4	118.86 (10)	C12—C11—H11B	110.6
O2—C4—N2	122.55 (10)	H11A—C11—H11B	108.7
O2—C4—C3	123.97 (11)	C17—C12—C13	118.85 (11)
N2—C4—C3	113.48 (10)	C17—C12—C11	120.61 (12)
N3—C5—C1	109.22 (9)	C13—C12—C11	120.53 (11)
N3—C5—C6	111.64 (9)	C14—C13—C12	120.51 (12)
C1—C5—C6	110.88 (9)	C14—C13—H13	119.7
N3—C5—C7	106.23 (9)	C12—C13—H13	119.7
C1—C5—C7	108.70 (9)	C13—C14—C15	120.27 (13)
C6—C5—C7	110.02 (10)	C13—C14—H14	119.9
C5—C6—H6A	109.5	C15—C14—H14	119.9
C5—C6—H6B	109.5	C16—C15—C14	119.59 (12)
H6A—C6—H6B	109.5	C16—C15—H15	120.2
C5—C6—H6C	109.5	C14—C15—H15	120.2
H6A—C6—H6C	109.5	C15—C16—C17	119.98 (12)
H6B—C6—H6C	109.5	C15—C16—H16	120.0
C5—C7—H7A	109.5	C17—C16—H16	120.0
C5—C7—H7B	109.5	C12—C17—C16	120.77 (13)
H7A—C7—H7B	109.5	C12—C17—H17	119.6
C5—C7—H7C	109.5	C16—C17—H17	119.6
			
C2—N1—C1—N2	−1.36 (16)	N1—C1—C5—C7	102.55 (12)
C2—N1—C1—C5	−178.04 (9)	N2—C1—C5—C7	−74.35 (12)
C4—N2—C1—N1	0.28 (18)	C9—O4—C8—O3	−5.78 (16)
C4—N2—C1—C5	177.10 (10)	C9—O4—C8—C2	171.97 (9)
C1—N1—C2—C3	0.47 (16)	C3—C2—C8—O3	4.06 (17)
C1—N1—C2—C8	−176.14 (10)	N1—C2—C8—O3	−179.15 (10)
N1—C2—C3—O1	−179.23 (10)	C3—C2—C8—O4	−173.74 (10)
C8—C2—C3—O1	−2.66 (18)	N1—C2—C8—O4	3.06 (14)
N1—C2—C3—C4	1.43 (17)	C5—N3—C10—O5	−11.21 (19)
C8—C2—C3—C4	178.00 (10)	C5—N3—C10—O6	170.86 (10)
C1—N2—C4—O2	−178.10 (11)	C11—O6—C10—O5	5.83 (18)
C1—N2—C4—C3	1.58 (16)	C11—O6—C10—N3	−176.18 (10)
O1—C3—C4—O2	−2.06 (18)	C10—O6—C11—C12	173.06 (10)
C2—C3—C4—O2	177.35 (11)	O6—C11—C12—C17	137.37 (11)
O1—C3—C4—N2	178.27 (10)	O6—C11—C12—C13	−43.44 (15)
C2—C3—C4—N2	−2.32 (16)	C17—C12—C13—C14	0.16 (18)
C10—N3—C5—C1	63.17 (13)	C11—C12—C13—C14	−179.05 (11)
C10—N3—C5—C6	−59.82 (14)	C12—C13—C14—C15	0.83 (19)
C10—N3—C5—C7	−179.76 (10)	C13—C14—C15—C16	−0.91 (19)
N1—C1—C5—N3	−141.95 (10)	C14—C15—C16—C17	0.00 (19)
N2—C1—C5—N3	41.15 (12)	C13—C12—C17—C16	−1.07 (17)
N1—C1—C5—C6	−18.51 (14)	C11—C12—C17—C16	178.14 (11)
N2—C1—C5—C6	164.59 (10)	C15—C16—C17—C12	1.00 (18)

### Hydrogen-bond geometry (Å, °)

**Table:**

*D*—H···*A*	*D*—H	H···*A*	*D*···*A*	*D*—H···*A*
N3—H3···O5^i^	0.882 (15)	2.133 (15)	2.8911 (14)	143.7 (12)
N2—H2···O2^ii^	0.938 (16)	1.886 (16)	2.8135 (16)	169.3 (13)
O1—H1···O3	0.918 (17)	1.788 (17)	2.6163 (14)	148.7 (16)

Symmetry codes: (i) *x*, −*y*+1/2, *z*+1/2; (ii) −*x*+1, −*y*, −*z*+2.
